# Potential role of endothelial progenitor cells in the pathogenesis and treatment of cerebral aneurysm

**DOI:** 10.3389/fncel.2024.1456775

**Published:** 2024-08-12

**Authors:** Jin Yu, Qian Du, Xiang Li, Wei Wei, Yuncun Fan, Jianjian Zhang, Jincao Chen

**Affiliations:** ^1^Department of Neurosurgery, Wuhan Asia General Hospital, Wuhan, Hubei, China; ^2^Department of Neurosurgery, Zhongnan Hospital of Wuhan University, Wuhan, Hubei, China; ^3^Department of Infectious Diseases, Zhongnan Hospital of Wuhan University, Wuhan, Hubei, China; ^4^Department of Respiratory and Critical Care Medicine, Laifeng County People’s Hospital, Enshi, Hubei, China

**Keywords:** endothelial progenitor cells, cerebral aneurysm, endothelial dysfunction, endothelialization, endovascular therapy

## Abstract

Cerebral aneurysm (CA) is a significant health concern that results from pathological dilations of blood vessels in the brain and can lead to severe and potentially life-threatening conditions. While the pathogenesis of CA is complex, emerging studies suggest that endothelial progenitor cells (EPCs) play a crucial role. In this paper, we conducted a comprehensive literature review to investigate the potential role of EPCs in the pathogenesis and treatment of CA. Current research indicates that a decreased count and dysfunction of EPCs disrupt the balance between endothelial dysfunction and repair, thus increasing the risk of CA formation. Reversing these EPCs abnormalities may reduce the progression of vascular degeneration after aneurysm induction, indicating EPCs as a promising target for developing new therapeutic strategies to facilitate CA repair. This has motivated researchers to develop novel treatment options, including drug applications, endovascular-combined and tissue engineering therapies. Although preclinical studies have shown promising results, there is still a considerable way to go before clinical translation and eventual benefits for patients. Nonetheless, these findings offer hope for improving the treatment and management of this condition.

## Introduction

Cerebral aneurysms (CA), also known as intracranial aneurysms, are abnormal focal dilations of the wall of cerebral arteries, typically occurring at branching points within the circle of Willis (Vlak et al., 2011; Brown and Broderick, 2014; Macdonald and Schweizer, 2017). These aneurysms are often saccular in shape, hence the term “berry aneurysms” (Brown and Broderick, 2014). CA occur in about 1–2% of the population, with prevalence estimates from imaging studies suggesting a frequency of 0.5–3% in the general population (Brown and Broderick, 2014; Tawk et al., 2021). They are more commonly found in women than men, with a ratio of 3:1, and are predominantly detected in the anterior locations of the circle of Willis (Sedat et al., 2005; Brown and Broderick, 2014; Abecassis et al., 2017). CA can lead to numerous clinical manifestations such as subarachnoid hemorrhage (SAH), compression of adjacent cerebral structures, causing symptoms like cranial nerve palsies, hemiparesis, field defects, seizures, brainstem compression, and transient ischemic attacks or cerebral infarction due to distal embolization (Brown and Broderick, 2014).

About 50% of CA are discovered following a SAH, which has a global incidence of approximately 6.1 per 100,000 person-years, with a prevalence of 8.09 million cases (Etminan et al., 2019; Tsao et al., 2023). The burden of SAH includes a high rate of mortality and long-term disability. About 13% of patients will die in the hospital of aneurysmal SAH, and up to 26% will die before reaching the hospital (Wahood et al., 2022). And of those who survive, many experience significant long-term disabilities., such as brain damage, hydrocephalus, and cognitive impairment (Wan et al., 2016; Toth and Cerejo, 2018). The economic impact is substantial, with inpatient hospital charges for patients with aneurysmal SAH reported to be $373,353.94 in the United States (Mualem et al., 2022).

CA is not a single disease entity: some can result from a genetic vulnerability, others from infection or inflammation. The pathophysiology of cerebral aneurysms involves complex interactions between genetic predisposition, hemodynamic stress, and vascular wall abnormalities. Factors such as hypertension, smoking, and genetic mutations contribute to the weakening of the arterial wall, leading to aneurysm formation and potential rupture (Brisman et al., 2006; Etminan and Rinkel, 2017; Frösen et al., 2019; Toader et al., 2023). And the functional deficiencies of cells involved in vascular wall remodeling, such as endothelial progenitor cells (EPCs) and smooth muscle cells (SMCs), may be crucial actors in the pathophysiology of CA (Lee et al., 2015; Xu et al., 2019).

The relationship between EPCs and CA is complex and multifaceted. EPCs are cells that originate from the bone marrow and migrate to areas of blood vessel damage, where they differentiate into functional endothelial cells (ECs) and contribute to the repair and maintenance of blood vessels integrity (Asahara et al., 1997). In patients with CA, the levels and function of circulating EPCs are altered, suggesting that these cells play a crucial role in the development and progression of these conditions (Liang et al., 2014). EPCs represent the essential initiating factor of re-endothelialization process that responded for aneurysm complete occlusion after endovascular treatment (Li et al., 2013, 2021). Targeted approaches that promote the mobilization of EPCs and increase their circulating count have demonstrated promising therapeutic effects in preclinical studies of CA (Liu et al., 2016a,b). Therefore, a deeper understanding of the relationship between EPCs and CA at the pathological level may provide valuable insights into the development and progression of these conditions and inform the development of new, more effective treatments. It is not optimistic that there is not a lot of research about EPCs and CA at present, which also suggests that a summary is urgently needed in this field to provide reference for future research direction.

In this narrative review, we aim to provide a comprehensive understanding of the involvement of EPCs in the pathogenesis of CA and to evaluate the potential of EPCs as a target for the prevention and treatment of intracranial aneurysms.

## The role of EPCs in endothelial function and integrity

The vascular endothelium acts as the first line of defense in protecting the vascular wall from direct stress caused by chemical, inflammatory, and mechanical factors (Aoki et al., 2011). Fortunately, our body has a natural mechanism for repairing damaged or dysfunctional endothelium, which involves the activation of various signaling pathways and the recruitment of repair cells (Evans et al., 2021). EPCs have been proven to play a crucial role in maintaining vascular homeostasis and function by promoting neovascularization and re-endothelialization of injured vessels (Sobhan et al., 2012). EPCs were first identified by Asahara et al. (1997) in isolated mononuclear cells from human peripheral blood (Asahara et al., 1997). These bone marrow (BM)–derived cells with high proliferative ability were found to have the potential to differentiate into ECs (Laurenzana et al., 2015) and are involved in both physiological processes triggered by endothelial dysfunction, such as wound healing and the ovarian cycle, as well as pathological events such as hypertension, stroke, and cancer (Schlager et al., 2011; de Cavanagh et al., 2014). EPCs are believed to originate from mesoderm cells during embryonic development, just like hematopoietic stem cells (De Val and Black, 2009). In a healthy state, EPCs reside in the BM microenvironment with low oxygen levels and high levels of stromal cell-derived factor 1 alpha (SDF-1α), which is essential for their survival (Balaji et al., 2013). However, during times of inflammation, injury, or hypoxia, EPCs are “mobilized” and leave the BM, entering the bloodstream due to triggers such as chemokines, matrix metalloproteinase (MMP) 9, vascular endothelial growth factor (VEGF), and nitric oxide (NO) (Velazquez, 2007; Tilling et al., 2009). Once they reach the target tissue, they are activated and guided by tissue-specific chemokine signaling. After adhering to the blood vessel and tissue repair sites through integrins, EPCs migrate through the endodermis to perform their function in neovascularization and re-endothelialization by transforming into ECs and remodeling the extracellular matrix components (Qin et al., 2006; Hynes, 2009). EPCs play a crucial role in maintaining the integrity of vascular endothelium by producing pro-angiogenic factors that boost the proliferation, survival, and function of mature ECs and other nearby progenitor cells, such as smooth muscle progenitor cells and SMCs (Laurenzana et al., 2015).

The formation of aneurysms is widely believed to result from an imbalance between the factors that damage the vascular endothelium and the mechanisms responsible for repairing this damage (Soldozy et al., 2019; Xu et al., 2019). Thus, exploring the different expressions of EPCs between CA patients and healthy people may open the window to gain insight into the mechanisms of CA pathogenesis.

## Current research on EPCs count and function in CA

In order to summarize the current knowledge on the relationship between EPCs and CA, A comprehensive literature search in PubMed, Embase and Ovid was performed. Search terms included all possible combinations of “Cerebral aneurysm,” “Intracranial aneurysm” and “Endothelial progenitor cell.” Only studies published in English or Chinese up to 1 April 2024, were considered. References in identified articles were also manually screened. This review was reported in accordance with the SANRA guidelines (Baethge et al., 2019). Several clinical and preclinical studies focused on EPCs characteristics in the CA condition have been found. Details of these studies can be found in Table 1.

**TABLE 1:** Details of studies about endothelial progenitor cells (EPCs) in cerebral aneurysm (CA).

References	Country	Samples	Methods of aneurysm modeling	EPCs identification standard	EPCs source	EPCs abnormalities in CA condition	Therapeutic exploration
Wei H. et al., 2011	China	56 CA patients (21 unruptured and 35 ruptured) and 40 healthy controls	–	CD34+/CD133+ or CD34+/VEGFR2+	Peripheral blood	Decreased: number of circulating EPCs, migratory capacity; Increased: cellular senescence	–
Xu et al., 2011	China	Rats	Unilateral common carotid artery and bilateral renal arteries surgically ligated + high-salt diet	CD34+CD133+	Peripheral blood	Decreased: number of circulating EPCs	EPO increased circulating EPCs, related to vascular remodeling, and the rate of CA formation and progression.
Wei H. J. et al., 2011	China	14 patients with ruptured CA	–	CD34+CD133+	Peripheral blood	Decreased: number of circulating EPCs	–
Fang et al., 2012	China	Rabbits	Porcine pancreatic elastase -induced	(VEGFR-2), CD133 and CD34	Bone marrow		EPC participated within the neointima formation after CA induction
Aronson et al., 2012	USA	Rabbits		PECAM-1/CD-31 and dii-Ac-LDL uptake	Carotid artery.		EPCs-seeded biopolymer endovascular implantation promotes confluent monolayer of endothelial cells with underlying neointima
Liang et al., 2014	China	24 patients with unruptured CA	–	CD34+CD133+KDR+	Peripheral blood	Decreased: EPCs count, proliferative, migratory and adhesive capacities	–
Li et al., 2014	China	Rats	Unilateral common carotid artery and bilateral renal arteries surgically ligated + high-salt diet	CD34, vwf, VE-cadherin, KDR	Human umbilical cord blood		ECFCs transfusion reduced vascular degeneration after CA formation
Li et al., 2014	China	Rabbits	Porcine pancreatic elastase -induced	CD34+CD133+KDR+	Bone marrow		SDF-1α promote reendothelialization CA induction by promoted endothelial-lineage cell mobilization
Gao et al., 2014	China	Rats	Unilateral common carotid artery and bilateral renal arteries surgically ligated	CD133, CD34, and VEGFR-2	Bone marrow		SDF -1α labeled coil accelerates CA organization and occlusion of neck remnant by recruiting endothelial progenitor cells
Li et al., 2015	China	Rats	Unilateral common carotid artery and bilateral renal arteries surgically ligated + high-salt diet	CD34+CD133+	Peripheral blood		Aspirin Inhibits Degenerative Changes of Aneurysmal Wall via promote EPCs Mobilization
Liu et al., 2016a	China	Rats	Arterial graft anastomosis	CD34+KDR+	Femur marrow		Rosuvastatin promote of aneurysm neck endothelialization after coil embolization by promotion of EPCs
Liu et al., 2016b	China	Rats	Arterial graft anastomosis	CD34+KDR+	Femur marrow		EPO increased the number of circulating EPCs and improved endothelialization After Coil Embolization
Gao et al., 2016	China	Rats	Unilateral common carotid artery and bilateral renal arteries surgically ligated	CD34+CD133+ KDR+	Bone marrow		SDF -1α labeled coil accelerates CA organization and occlusion of neck remnant by recruiting endothelial progenitor cells
Marosfoi et al., 2017	USA	Rabbits	Elastase-induced	CD34+	Aneurysm lumen		–
Li et al., 2017	China	Rabbits	Unilateral common carotid artery and bilateral renal arteries surgically ligated	CD34+CD133+ KDR+	Bone marrow		Intravenous injection of rhdf-1α can accelerate the re-endothelialization of the neck of aneurysm after flow divert device implantation by increasing EPCs.
Wei et al., 2019	China	Rats	Unilateral common carotid artery and bilateral renal arteries surgically ligated + high-salt diet	CD34+CD133+	Peripheral blood		Atorvastatin increases the number of EPCs in circulation to inhibit endothelial injury and aneurysm formation
Yu et al., 2019	China	Rats	Arterial bag anastomosis	CD34+KDR+	Femur marrow		mir-31a-5p improved endothelialization via elevated the number of circulating EPCs
Xu et al., 2021	China	Rats	Left common carotid artery ligation	CD34+CD133+	Peripheral blood		Simvastatin increases the number of EPCs in circulation to inhibit aneurysm formation

EPO, Erythropoietin; ECFC, endothelial colony-forming cells; SDF -1α, stromal cell-derived factor-1α.

### Human research

Direct evidence from human CA clinical samples confirmed abnormal count and dysfunction of EPCs in the CA condition. For example, in Wei H. et al. (2011) described a decreased number and impaired migratory capacity of EPCs from peripheral blood in both unruptured (21 patients) and ruptured (35 patients) CA patients compared with healthy control subjects. Consistently, data from Liang et al. (2014) indicated the EPCs count was also significantly decreased in 24 unruptured CA patients, accompanied by decreases in proliferative, migratory and adhesive capacities, compared with EPCs from the control group. These findings seem to be contrary to our conventional understanding that a larger number of EPCs should be sent into circulation after aneurysm formation to repair damaged vascular intima. This discrepancy may be due to the fact that the same factors that cause harm to the vascular intima–such as trauma or blood flow shear stress–also hinder the mobilization or activation of EPCs (Dzau et al., 2005; Zampetaki et al., 2008). Wei H. J. et al. (2011) measured numbers of circulating EPCs in CA patients before and after surgical coil embolization, and they found a biphasic change pattern of circulating EPCs, which first decreased upon admission (before surgery) and then increased after surgery as compared to healthy subjects. They proposed that the mechanism of reducing circulating EPCs after CA induction may be related to consumption and stress induced by injury, similar to a phenomenon in the early hours of traumatic brain injury (Liu et al., 2007).

### Translational (animal and *in vitro*) research

**FIGURE 1:**
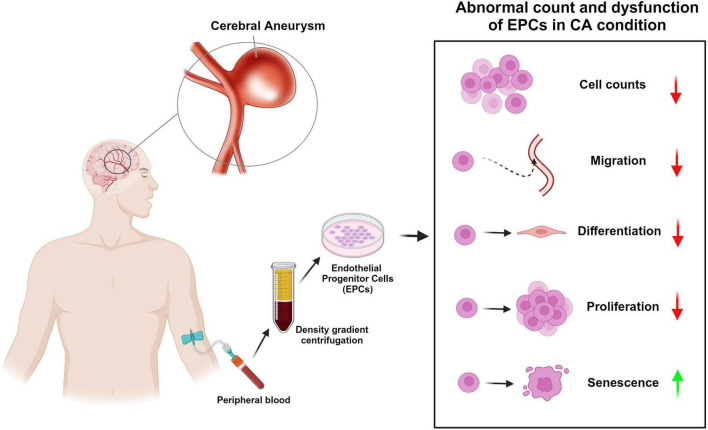
Abnormal count and dysfunction of endothelial progenitor cells (EPCs) in cerebral aneurysm (CA) condition. This diagram shows the rough process of obtaining EPCs from peripheral blood of CA patients and summarizes the abnormal cell counts and dysfunction of EPCs in CA condition reported in previous studies. The red arrow indicates “decreases” and the green arrow indicates “increase.”

In translational research, animal models and *in vitro* studies have also explored EPCs in the context of CA. When researchers created animal models of CA by surgically ligating intracranial arteries to cause hemodynamic damage to the intima, decreased EPCs levels was detected only in rats that underwent surgery, rather than non-surgical control rats (Xu et al., 2011). These findings suggest that the same factors that cause endothelial damage—such as hemodynamic stress—also impede the mobilization or activation of EPCs. Further supporting this, *in vitro* studies have shown that the levels and functionality of EPCs are altered in CA patients, which may be due to increased EPCs senescence (Leone et al., 2009). For example, Wei H. et al. (2011) found that EPCs isolated from CA patients showed higher levels of senescence-associated β-galactosidase activity, a marker for cell senescence, than those from normal subjects. This increased senescence activity was significantly negatively correlated with the number of circulating EPCs, indicating that EPCs senescence might play a role in the reduced EPCs count observed in CA conditions (Figure 1 and Table 1).

## Pre-clinical EPCs-based treatment research for CA

Above findings suggest that the formation of CA is related to both a decrease in the number of circulating EPCs and a decrease in their biological abilities. These findings naturally raise the question of whether reversing the abnormalities of circulating EPCs in CA patients could repair the intima or stabilize aneurysms.

To test this hypothesis, Fang et al. (2012) performed an elastase-induced approach (Wang et al., 2009) to simulate the destruction of the vascular intima during aneurysm formation to induce carotid saccular aneurysms in rabbit models. Then, the autologous fluorescent labeled EPCs were injected into the animals in either the *in situ* carotid artery (ISCT group) or marginal ear veins (IVT group). Three weeks after undergoing elastase treatment, the EPCs-transfusion group animals were found to have thicker aneurysmal vessel walls and more ECs and a less-disrupted internal elastic lamina compared to controls. Fluorescein–labeled EPCs were found in both the thrombosis and neointima of the aneurysm wall in 5/5 animals in the ISCT group and in 3/5 animals in the IVT group. These results suggested that EPCs are required in the repair response to vascular injury after aneurysm formation. However, labeled EPCs in this study were not found at the intimal surfaces of the aneurysms, and immunohistochemical staining showed that there was no ECs growth in the neointima. Since EPCs differentiation depends on the microenvironment provided, the authors proposed that the inflammatory microenvironment of injured vessels may have suppressed EPCs from differentiating into the epithelium in this rabbit model. Besides, this study didn’t show whether the transfusion of EPCs could reduce the size of the aneurysm or the risk of aneurysm rupture. Previous studies demonstrated that the degeneration of the vessel wall and subsequent inflammation invasion followed by vascular remodeling and aneurysm enlargement are associated with CA prone to rupture (Kataoka et al., 1999; Aoki et al., 2008). In this regard, Li et al. (2014) conducted a study to see if the supplementation of EPCs could reverse vascular degeneration by regulating the inflammation cascade and apoptosis activity. Endothelial colony-forming cells (ECFCs), a unique population of EPCs that possess high proliferation, self-renewal and *in vivo* vasculogenic potentials (Ingram et al., 2004), were adopted in this study. Briefly, CA was generated in rats via arterial surgical ligation, and ECFCs were then intravenously injected. At 2 months after aneurysm induction, the aneurysm vessel was harvested for pathological, immunological and molecular biological tests. Interestingly, ECFCs transfusion significantly reduced the degeneration of the internal elastic lamina (IEL), media thinning and the CA size. ECFCs effectively inhibited the MMP-driven wall destruction by downregulating MMP-2 and MMP-9 expression and upregulating the tissue inhibitor of metalloproteinases-1 (TIMP-1). Furthermore, inflammation factors like vascular cell adhesion molecule-1 (VCAM-1) and NF-κB and macrophage infiltration in the aneurysm wall were also suppressed by ECFCs transfusion.

Besides the direct transplantation of EPCs, various factors stimulating the mobilization and recruitment of EPCs to the site of an aneurysm were applied to test the potential role of EPCs in CA repair. Erythropoietin (EPO) is believed to promote stem cell mobilization, differentiation and maturation toward cells of endothelial lineage (Burger et al., 2006). Xu et al. (2011) treated CA rats EPO and found that EPO treatment significantly increased the numbers of circulating EPCs, reduced aneurysm volume, decreased IEL score, and thickened medial layer at 1 month and 3 months after surgery as compared to rats without EPO treatment. Similarly, SDF-1α is a vital inflammation cytokine, and is also related to mobilizing progenitor cells and stem cells into injured tissues (Ceradini et al., 2004; Wang et al., 2008). Li et al. (2018) found the *in vivo* administration of SDF-α to rabbits with saccular aneurysms promoted endothelial-lineage cell mobilization into the peripheral blood and reendothelialization of the aneurysm wall. Hoh et al. (2014) also found that SDF-1 promotes endothelial cell and macrophage migration complied with robust angiogenesis in the walls of aneurysms. However, they accidentally found that mice given an anti-SDF-1 blocking antibody developed significantly fewer intracranial aneurysms. This infers that SDF-1 can promote endothelial repair by inducing endothelial cell migration and capillary tube formation, while it can also facilitate aneurysm formation and rupture by promoting angiogenesis and inflammatory cell infiltration of the aneurysm wall. This finding suggests that the role of EPCs in the pathogenesis of aneurysms is complex and multifaceted.

**FIGURE 2:**
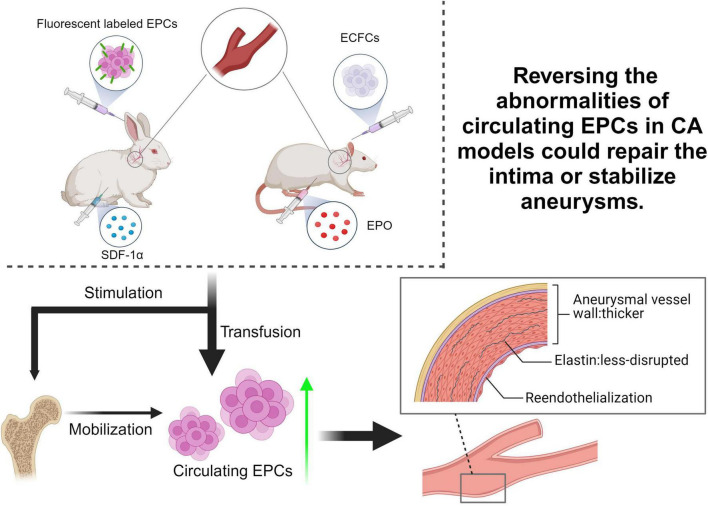
Summary of pre-clinical EPC-based treatment researches for CA. Current studies primarily used rabbit or rat as animal models to simulate CA, aiming to verify the hypothesis that reversing the abnormalities of circulating EPCs in CA patients could repair the intima or stabilize aneurysms. By transfusing EPCs or factors stimulating EPCs mobilization, such as Stromal Cell-Derived Factor 1 alpha (SDF-1α) and Erythropoietin (EPO), into CA animal models to increase circulating EPCs, the progression of vascular degeneration after aneurysm induction was effectively reduced. ECFCs, endothelial colony-forming cells.

Taken together, the above findings demonstrated that the transfusion or stimulation of EPCs was effective in reducing the progression of vascular degeneration after aneurysm induction in animal models, which implies EPCs are a promising target for the development of new therapeutic strategies to facilitate CA repair (Figure 2 and Table 1). It is worth noting that these studies are currently preclinical animal studies, and clinical studies involving humans are still lacking.

## The effect of current and emerging treatments for CA on EPCs

The involvement of EPCs in the formation and repair of aneurysm has inspired researchers to consider new therapeutic approaches for treating CA based on EPCs. This involves using agents to regulate the quantity/function of EPCs in the patient’s circulation to enhance the process of repairing the vascular intima and stabilize the aneurysm, or even treat it.

### Drugs stimulating EPCs mobilization

One common approach explored in published studies is the use of clinical drugs to stimulate the mobilization and recruitment of EPCs to the site of an aneurysm. For example, pre-clinical animal experiments demonstrated that statins such as rosuvastatin and atorvastatin can promote the mobilization of EPCs, leading to enhanced aneurysm neck endothelialization and reduced degeneration of aneurysms (Wei et al., 2019; Xu et al., 2021). Similarly, aspirin, a well-established antiplatelet and nonsteroidal anti-inflammatory drug, was also proven to significantly reduce the degeneration of aneurysm walls in a rat model by augmenting EPCs mobilization and inhibiting macrophage-mediated chronic vascular inflammation (Li et al., 2015). It is worth noting that both statins and aspirin, in addition to increasing the number of EPCs, also have the effect of suppressing inflammation in the aneurysm wall. The expression of many inflammatory factors such as iNOS, MMP-2, MMP-9, VEGF, NF-κB and macrophage chemoattractant protein 1 (MCP-1) is inhibited by these drugs. Previous studies have demonstrated that after sustained hemodynamic shear stress causes endothelial dysfunction at the bifurcation, inflammation is activated and extensive invasion of macrophages, T lymphocytes, and B lymphocytes occurs in aneurysm tissue, leading to increased damage to the vessel wall (Chyatte et al., 1999; Deanfield et al., 2007). Therefore, these drugs with the dual effect of activating EPCs and anti-inflammation may be more effective in stabilizing aneurysm walls and preventing aneurysm rupture than factors with the sole effect of EPCs elevating.

### Endovascular surgery combined with EPCs agonists

In addition to drug applications, novel tissue engineering therapies of “endovascular surgery combined with EPCs agonists” also show promising prospects in CA treatment (Ravindran et al., 2019). After coil embolization, the neointima and endothelium are formed in the neck of CAs to seal the aneurysm cavity in order to prevent hemodynamic stress from the parent artery toward the aneurysm wall (Sugiu et al., 1995; Berenstein et al., 2009). Animal research has revealed that recanalization of previously closed aneurysms occurs only when there is insufficient endothelialization covering the ostium (Reul et al., 1997; Macdonald et al., 1998; Kallmes et al., 1999; Li et al., 2013). This has also been supported by autopsy and clinical studies conducted on humans, which have linked recanalization to inadequate endothelialization of the aneurysm ostium (Molyneux et al., 1995; Mizoi et al., 1996). Therefore, the use of EPCs activators to enhance endothelialization over the aneurysm lumen / neck after endovascular therapy seems reasonable, and this approach has indeed shown promising clinical application in animal studies. For example, the combination of the stimulation of EPCs by agonists such as miR-31a-5p (Yu et al., 2019), EPO (Liu et al., 2016b) and rosuvastatin (Liu et al., 2016a) before or after with endovascular surgery have shown promising results in the treatment of aneurysms in rats model. By inducing the process of endothelialization in the neck of an aneurysm after coil embolization, these approaches can reduce the risk of aneurysm rupture and provide a safer and more effective treatment option for patients.

As mentioned before, SDF-1α is a vital chemoattractant to stem cells and potentially facilitates reendothelialization. Gao et al. (2014) used the SDF-1α-coated coils in CA endovascular occlusion and found better organized fibrous tissue bridging the orifice of aneurysms on days 14 and 28 after coil implantation in SDF-1α-coated coils + EPCs transplantation rats than in the unmodified coils rats’ group. They suggested that SDF-1α-coated coils with EPCs or mesenchymal stem cells transplantation may be beneficial in the aneurysm healing and endothelialization at the orifice of the embolized aneurysm (Gao et al., 2016).

### Flow diverter (FD) implants and EPC-based tissue engineering approaches

Besides coils, high rates of complete aneurysm occlusion have been reported using FD implants that lead to parent vessel reconstruction and intrasaccular aneurysm thrombosis. According to Marosfoi et al. (2017), circulating CD34+ EPCs contribute to endothelialization after FD implantation in rabbit model and that EPCs are present throughout the healing process up to 60 days. Preliminary evidence demonstrated the temporal and spatial dependence of endothelialization on FD design. Special FD designs can incorporate these analyses during development to accelerate the in-situ tissue response to the scaffold. What’s more, the joint use of recombinant human SDF-1α administration accelerates endothelial-like cells expansion at the aneurysm neck in elastase-induced saccular aneurysm rabbits after FD treatment (Li et al., 2017). In addition, a novel tissue engineering approach using an endothelial progenitor cell-seeded biopolymer to treat intracranial saccular aneurysm has been proposed (Aronson et al., 2012). In this study, EPCs cultures were trypsinized and combined with the fibrin polymer. The fibrin + EPCs polymer was then injected into the rabbits’ aneurysm via the microcatheter. In aneurysms treated with endovascular fibrin + EPCs, a confluent monolayer of ECs with underlying neointima was demonstrated across the neck at 16 weeks post-treatment, which was not observed with aneurysms treated using the other methods such as surgical clipping, coil and fibrin polymer treatment alone (Aronson et al., 2012).

**FIGURE 3:**
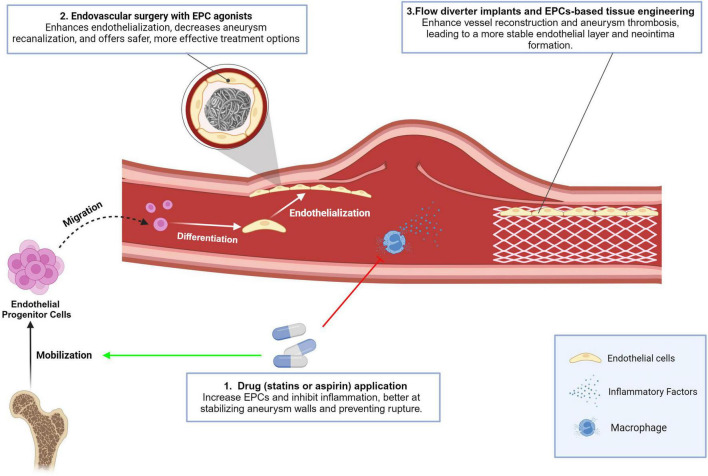
Conclusion in the effect of current and emerging treatments for CA on EPCs. Drugs stimulating EPCs mobilization: Statins (rosuvastatin, atorvastatin) and aspirin mobilize EPCs to aneurysm sites, enhancing endothelialization of aneurysm necks, reducing degeneration, and suppressing inflammation by inhibiting factors like iNOS, MMP-2, MMP-9, VEGF, NF-κB, MCP-1, and VCAM-1. These drugs also offer anti-inflammatory benefits, aiding aneurysm stabilization and rupture prevention; Endovascular surgery with EPCs agonists: EPCs activators enhance endothelialization over aneurysm areas after surgery. Combining EPCs stimulation with agonists (e.g., miR-31a-5p, EPO, rosuvastatin) improves treatment outcomes by reducing recanalization and enhancing safety and effectiveness; Flow diverter implants and EPCs-based tissue engineering: Flow diverter implants facilitate vessel reconstruction and aneurysm thrombosis, with EPCs supporting endothelialization. EPC-based tissue engineering uses biopolymer- combined EPCs injected into aneurysms, resulting in a stable endothelial layer and neointima formation.

Briefly, the role of EPCs in the formation and repair of cerebral aneurysms has gained increasing attention in recent years. Various therapeutic approaches have been explored to utilize EPCs for the treatment of CA, including drug applications with / without endovascular surgery, and novel device and tissue engineering therapies. These novel therapeutic approaches have provided new insights into the treatment of CAs and offer promising possibilities for patients in the future (Figure 3 and Table 1). It is important to note that these findings are based on preclinical animal studies, and there is still a lack of clinical studies involving humans.

### Future directions

The existing problems will guide the future direction. First of all, there is a scarcity of relevant studies, particularly those investigating the expression characteristics of EPCs in clinical samples of patients with CA, and the sample sizes in each study are relatively small. As a result, the relevant findings may be subject to considerable deviation. For example, the studies failed to clarify the exact role of EPCs subtypes in the development of CA. As we know, EPCs can be categorized as “early EPCs” or “late EPCs” based on their morphology and function. Early EPCs are characterized by their ability to form clusters or colonies *in vitro*, while late EPCs are more similar to mature ECs in phenotype and exhibit tubular formation and proliferative capabilities (Hur et al., 2004; Shantsila et al., 2007). These differences are identified through different culture methods and various cell surface markers, such as CD34, CD45, CD133, and VEGFR-2 (Yu et al., 2020). In the reviewed studies, researchers have not made a clear distinction between the two EPC subtypes, and have mainly used CD34+/CD133+ with or without VEGFR2+ to define “circulating EPCs.” Although these studies have produced consistent results regarding the decreased number and function of EPCs in CA patients, it is important to distinguish between the expression characteristics of EPC subtypes in CA, as different subtypes may exhibit opposite characteristics in the same disease. For instance, in Moyamoya disease, another cerebrovascular disease with progressive vascular stenosis associated with intracranial abnormal angiogenesis, a decrease in the number and reduced proliferation ability of early EPCs was observed, but late EPCs presented a completely opposite characteristic (Jung et al., 2008; Rafat et al., 2009). In fact, different subtypes of EPCs also show different effects on CA repair in the CA. for example, ECFCs, a “late EPCs,” is considered to have strong proliferation ability and shows significantly better arterial wall repair ability than unscreened circulating EPCs (Fang et al., 2012; Li et al., 2014). Future studies should use targeted cell markers to describe the expression characteristics of different EPCs subtypes in CA patients and explore the different contributions of early / late EPCs to the pathogenesis of CA.

Second, the studies included in this paper lack comprehensive investigation into the mechanisms through which EPCs affect CA formation or repair. Although some studies have identified related factors such as VEGF, MMP, and iNOS, the specific mechanisms by which these factors regulate EPCs have not been explored. Given the complexity of organisms, the emergence of EPCs may merely represent one of many reactions that can occur when drugs or implants are introduced. For example, statins lower blood lipids, aspirin inhibits inflammation, and coils can cause thrombosis. Thus, reduced vascular degeneration following aneurysm induction cannot be solely attributed to EPCs, and these factors likely participate in vascular repair through alternative mechanisms. Similarly, the conditions, dosages, side effects, and long-term effects of drugs and new tissue engineering materials have yet to be assessed through long-term, rigorous experiments. We anticipate the participation of more medical centers in future research on this topic, as well as additional exploration of the molecular mechanisms underlying EPCs regulation of CA formation, the standardization of drug/device usage, and an examination of side effects.

## Summary

### Mechanistic link between EPCs and CA

The involvement of EPCs in the pathogenesis of CA is primarily linked to their role in vascular repair and endothelial regeneration. EPCs are crucial for maintaining vascular integrity through neovascularization and re-endothelialization. Their dysfunction or reduced presence can exacerbate endothelial damage, which is a key precursor to aneurysm formation (Mandelbaum et al., 2013). This imbalance between endothelial damage and repair mechanisms, exacerbated by factors such as high wall shear stress and chronic inflammation, contributes to aneurysm pathogenesis (Soldozy et al., 2019; Xu et al., 2019). High shear stress leads to endothelial dysfunction, promoting inflammation and matrix degradation, processes that EPCs normally counteract through their reparative functions (Tutino et al., 2014; Zhou et al., 2014; Frösen et al., 2019; Koseki et al., 2020). Referring to the “Current research on EPCs count and function in CA” section we mentioned above, there is evidence that EPCs play an important role in both the occurrence and progression of CA due to their role in endothelial repair and their interaction with inflammation and stromal remodeling processes.

### Potential as a treatment strategy

Recent pre-clinical and clinical research has explored the potential of EPCs as a therapeutic target for CA. Various strategies have been employed to enhance EPCs function or mobilization, such as drug treatments and novel surgical techniques. Drugs like statins and aspirin have been shown to mobilize EPCs and simultaneously reduce inflammation within aneurysm walls, thus stabilizing aneurysms and reducing the risk of rupture (Li et al., 2015; Wei et al., 2019; Xu et al., 2021). Endovascular surgeries combined with EPCs agonists, such as EPO and SDF-1α, have demonstrated promise in enhancing endothelialization and reducing aneurysm recanalization (Liu et al., 2016a,b; Yu et al., 2019). Additionally, EPC-based tissue engineering approaches, including the use of EPC-seeded biopolymers and flow diverter implants, have shown improved outcomes in pre-clinical models, suggesting a significant therapeutic potential (Aronson et al., 2012; Li et al., 2017; Marosfoi et al., 2017).

### Integration with existing models of CA pathogenesis

The evidence linking EPCs to CA fits well into existing models of aneurysm formation, which emphasize the balance between endothelial damage and repair. EPCs play a pivotal role in counteracting endothelial dysfunction and promoting vascular repair, which aligns with the understanding that aneurysm formation involves a disruption of this balance. Current models of CA pathogenesis focus on the interplay between mechanical stress, inflammation, and endothelial dysfunction. EPCs, by influencing these processes, provide a crucial link between endothelial damage and the potential for therapeutic intervention. This mechanistic insight supports the integration of EPCs-targeted therapies within these models, highlighting their role in not only understanding CA development but also in developing effective treatments (Frosen et al., 2006; Aoki et al., 2011).

### Strengths and limitations

This review provides a comprehensive examination of the role of EPCs in CA pathogenesis and treatment, synthesizing evidence from clinical, preclinical, and translational research; it summarized various therapeutic approaches, including drug-based and tissue engineering strategies, showing promising results in preclinical models; and it discusses the potential of EPCs as a target for novel treatments, offering new insights into CA management.

However, the review also has limitations: it relies on a restricted literature search from specific databases, potentially missing relevant studies; Due to the limited number of relevant studies, most of which focus on preclinical animal research with limited clinical validation, the conclusions and inferences drawn from this review may not fully reflect the human condition; Many studies included have small sample sizes, which may introduce bias and limit generalizability.

## Conclusion

In conclusion, this paper has provided a detailed overview of the current understanding of the role of EPCs in the pathogenesis and treatment of CAs. Clinical and preclinical studies consistently show a decrease in both the number and function of EPCs in CA patients, which impairs the repair response to vascular injury and may contribute to aneurysm formation and progression. Preclinical studies using EPCs-based therapies, such as EPCs transfusion and drugs that stimulate EPCs mobilization, have demonstrated potential benefits in enhancing aneurysm neck endothelialization, reducing aneurysm wall degeneration, and stabilizing aneurysms. Specifically, drugs like statins and aspirin, as well as tissue engineering approaches combining EPCs with endovascular surgery, have shown promise in animal models. However, these findings are primarily from preclinical studies, and there is a lack of clinical trials to validate these approaches in human patients. Future research should focus on distinguishing the roles of different EPCs subtypes in CA, understanding the molecular mechanisms underlying EPCs-mediated repair, and conducting rigorous clinical trials to assess the safety and efficacy of EPC-based therapies in CA treatment. Despite these challenges, the promising results from preclinical studies suggest that targeting EPCs could be a viable strategy for improving the management and treatment of CA.

## Author contributions

JY: Conceptualization, Data curation, Formal analysis, Funding acquisition, Investigation, Methodology, Project administration, Resources, Software, Supervision, Validation, Visualization, Writing – original draft, Writing – review & editing. QD: Writing – original draft, Writing – review & editing. XL: Writing – review & editing, Conceptualization, Visualization, Software. WW: Writing – review & editing, Conceptualization, Visualization, Software. YF: Writing – original draft, Writing – review & editing. JZ: Writing – original draft, Writing – review & editing. JC: Conceptualization, Data curation, Formal analysis, Funding acquisition, Investigation, Methodology, Project administration, Resources, Software, Supervision, Validation, Visualization, Writing – original draft, Writing – review & editing.
